# *Micrometam C* Protects against Oxidative Stress in Inflammation Models in Zebrafish and RAW264.7 Macrophages

**DOI:** 10.3390/md13095593

**Published:** 2015-08-28

**Authors:** Hao Tang, Hui Ge, Zhi-Bin Chen, Xiong-Ming Luo, Feng-Juan Su, Yan-Bing Liang, Zhen-Yu Li, Jing-Guo Wu, Qing Yang, Li-Jin Zeng, Zhong-Fu Ma

**Affiliations:** 1 Department of General Internal Medicine, The First Affiliated Hospital, Sun Yat-sen University, Guangzhou 510080, China; E-Mails: kyrintang@163.com (H.T.); psyczb@aliyun.com (Z.-B.C.); xuyw8591@126.com (Y.-B.L.); leezhenyu99@163.com (Z.-Y.L.); wjg114500@163.com (J.-G.W.); yangqin804@163.com (Q.Y.); 164555182@qq.com (L.-J.Z.); 2 Department of Health Care Clinic, The First Affiliated Hospital, Sun Yat-sen University, Guangzhou 510080, China; E-Mail: gehui2009gh@126.com; 3 CAS Key Laboratory of Tropical Marine Bio-resources and Ecology, South China Sea Institute of Oceanology, Chinese Academy of Sciences, Guangzhou 510301, China; 4 Department of Neurology, The First Affiliated Hospital, Sun Yat-sen University, Guangzhou 510080, China; E-Mail: jisufengjuan@163.com

**Keywords:** *micrometam C*, inflammatory, zebrafish, NF-κB, NADPH oxidase

## Abstract

*Micrometam C* is a core of novel marine compound isolated from the mangrove associates *Micromelum falcatum.* In this study, we investigated the protective effects of *micrometam C* in inflammation models in the transgenic zebrafish line Tg (corola: eGFP) and RAW264.7 macrophages. We found that *micrometam C* significantly suppressed the migration of immune cells in tail-cutting-induced inflammation in transgenic zebrafish and reduced lipopolysaccharide (LPS)-induced reactive oxygen species (ROS) in both zebrafish and macrophages. In addition, *micrometam C* also restored LPS-induced reduction of endogenous antioxidants, such as catalase (CAT), glutathione (GSH) and superoxide dismutase (SOD). The protective effects of *micrometam C* were in parallel to its inhibition of NADPH oxidase and nuclear factor-kappa-binding (NF-κB) activity. Thus, the present results demonstrate that *micrometam C* protects against LPS-induced inflammation possibly through its antioxidant property.

## 1. Introduction

Inflammation is a defense response of body to injury and infection. Normally, inflammation protects body tissues against original insult through elimination of cause of cell injury and initiation of tissue repair. However, inappropriate or uncontrolled inflammations induce overactivity of the body and are harmful to the body [1,2,3]. Indeed, inflammation is major component of a wide variety of diseases, whereas anti-inflammation drugs, such as aspirin and other non-steroidal anti-inflammatory drugs (NSAIDs), can inhibit excessive inflammation and are beneficial in inflammation-related diseases, such as inflammatory bowel disease [4,5]. However, the gastrointestinal and cardiovascular side effects limit the clinical application of the current anti-inflammation drugs [6]. Thus, development of novel, more efficient, and safer anti-inflammatory drugs is still a major task.

LPS, the major component of the Gram-negative bacterial cell wall, can trigger and accelerate an inflammatory cascade. LPS has been linked to a wide variety of human diseases, including gastro-intestinal illness [7]. Thus, LPS has been widely used to mimic features of inflammatory diseases [8]. Reactive oxygen species (ROS), particularly generated from NADPH oxidase by immune cells, such as macrophages, is an important innate immune response to LPS. On the other hand, excessive ROS has strong pro-inflammatory effects and can serve as secondary messenger to further amplify LPS-induced inflammatory cascade [9].

The zebrafish is one of the most widely used vertebrate models [10,11]. Zebrafish has a fully developed immune system and are able to produce an inflammatory response when exposed to LPS. Therefore, the LPS zebrafish model provides an excellent platform for screening anti-inflammation drugs and studying their anti-inflammation mechanisms.

Small molecules from marine environment are an attractive alternative for anti-inflammatory drug discovery because the large number of bioactive, structurally diverse secondary metabolites has been found in marine environment [12,13]. Micrometams B–C were isolated from the mangrove associates *Micromelum falcatum* (Lour.) Tan. (5.5 kg), collected in Wenchang, Hainan Province, which were described in the experimental part of the literature [14].

The aim of the present study was to evaluate the effects of *micrometam C* on oxidative stress-associated inflammation and explore its protective mechanism in zebrafish *in vivo* and mouse macrophage RAW264.7 cells *in vitro*.

## 2. Results and Discussion

### 2.1. The Isolation and Preparation of Micrometam B and Micrometam C

**Chart 1:**
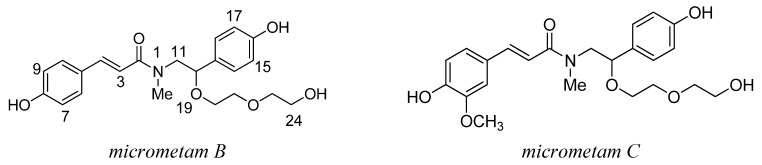
Chemical structure of *micrometam B and micrometam C*.

Micrometams B–C were isolated from the mangrove associates *Micromelum falcatum* (Lour.) Tan. (5.5 kg), collected in Wenchang, Hainan Province. The isolation of Micrometams B–C was conducted as previously described [14]. The structures of the isolated compounds were established as Chart 1 by analysis of spectroscopic data, including the MS, UV, IR, and NMR spectra.

### 2.2. Effect of Micrometam C in Tail Cutting-Induced Inflammation in Zebrafish Model

**Figure 1:**
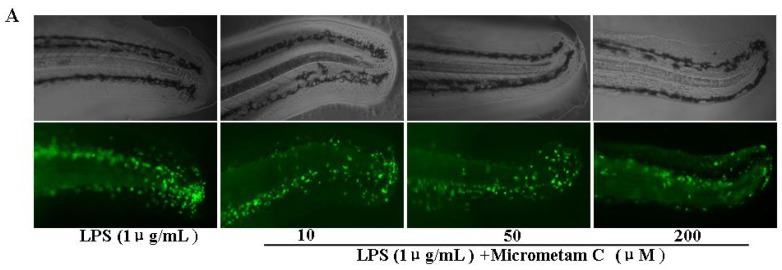

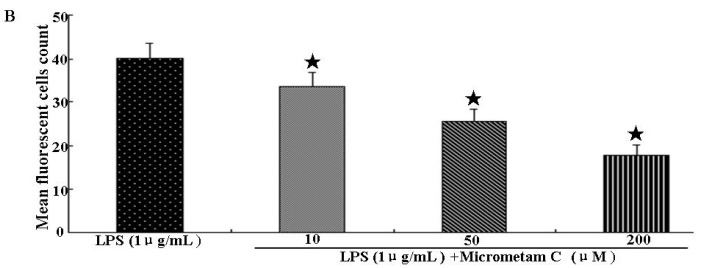
Effect of *micrometam C* in inflammation-induced zebrafish model by tail-cutting. Experiments were performed in triplicate and the data are expressed as mean ± SEM. Fluorescent cells count are significantly different at *p* < 0.05 as analyzed by ANOVA. (* *p* < 0.01 *vs.* LPS).

Considerable infiltration of phagocytes into the body tissue is one of pathological features in inflammation. The enhanced migration and activation of phagocytes are involved in the tissue injury during active stage of inflammation. Phagocytes can be primed by several stimulators, such as LPS of intestinal bacteria. Primed phagocytes then generate excessive ROS, which in turn causes oxidative mucosal damage. Given that the innate and acquired immune systems are conserved between zebrafish and humans, zebrafish is a good *in vivo* model for anti-inflammatory drug discovery. In the present study, we examined the anti-inflammatory action of *micrometam C* in transgenic Tg (coro1a:eGFP) zebrafish. In these transgenic animals, their macrophages and neutrophils are labeled with enhanced green fluorescent protein, which permits non-invasive and dynamic imaging the inflammation *in vivo* in transparent embryos and larvae. To induce the migration of phagocytes to a site of injury, we performed a transection of the tail in zebrafish larvae. As soon as tail-cutting was completed, the zebrafish larvae were incubated with tested compounds at final concentration 200 μM for 6 h. The mean numbers of fluorescent cells in tail-cutting-stimulated larvae with treatment of 1 μg/mL LPS was 40.1 % ± 3.5% whereas the mean numbers of fluorescent cells in tail-cutting-stimulated embryos were 23.7% ± 2.7% at *micrometam* *B* and 17.6% ± 2.6% at *micrometam C*. Given that *micrometam C* had stronger anti-inflammatory action than *micrometam B*, *micrometam C* was used in the further bioactivity evaluation. In the dose response experiments, *micrometam C* at 10, 50 and 200 μM dose-dependently decreased the mean numbers of fluorescent cells (Figure 1). Thus, the present study demonstrates that treatment with *micrometam C* at 10, 50 and 200 μM can dose-dependently inhibit tail-cutting-induced migration of immune cells towards the injured tail region in the larvae.

### 2.3. Effect of Micrometam C on Survival Rate, Heart Beat Rate and Morphological Changes in Zebrafish

**Figure 2:**
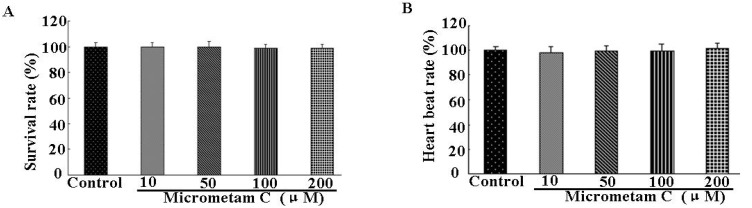
Effects of *micrometam C* on (**A**) survival rate (**B**) heartbeat rate of zebrafish embryo. Survival rate after treatment with different concentrations of the *micrometam C* was measured at 5 days postfertilization (dpf). The average heartbeat rate per minute of *micrometam C* treated embryos was recorded at 3 dpf. Experiments were performed in triplicate and the data are expressed as mean ± SEM. Survival rate and heartrate are not significantly different among different groups.

**Figure 3:**
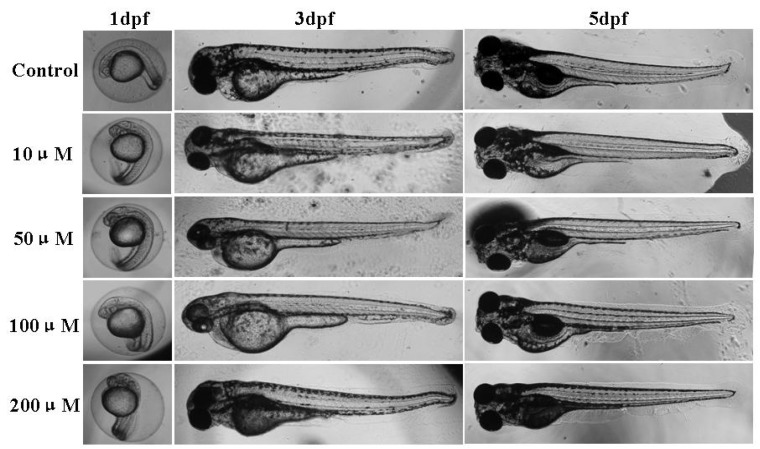
Effects of *micrometam C* on morphological changes of zebrafish embryo. Representative photographs of the morphological changes of the embryos treated with different concentrations of the *micrometam C* were taken. Experiments were performed in triplicate Morphological changes are not different among different groups.

**Figure 4:**
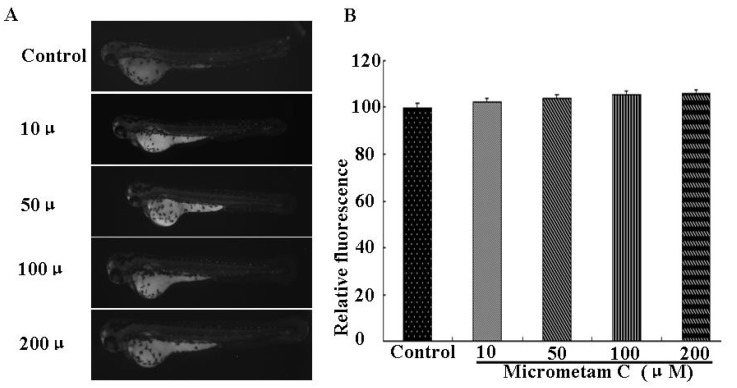
Effect of *micrometam C* on cell death in zebrafish embryo. (**A**) The cell death was measured after acridine orange staining by image analysis and fluorescence microscope; (**B**) The cell death was quantified using an Image J program. Experiments were performed in triplicate and the data are expressed as mean ± SEM. Cell deaths are not significantly different among different groups.

The survival rate, heartbeat rate and morphological changes were further examined in zebrafish embryos receiving *micrometam C* to determine the toxicity of *micrometam C*. There were no significant change in survival, heart beat rates in zebrafish receiving *micrometam C* at concentrations between 10 and 200 μM compared to the controls, indicating that there is no cardio-toxicity at the tested concentrations (Figure 2A). Although a slight increase in the heart beat rate was observed at the concentration of 200 μM (Figure 2B), the increase was not significant compared to the controls. In morphological assay, the tested concentrations between 10 to 200 μM did not induce any toxic effects on the developmental stages of the zebrafish embryo (Figure 3). The effect of *micrometam C* on cellular toxicity was further evaluated by means of cell death in zebrafish embryos. Cell death was not significantly increased at concentrations between 10 and 200 μM compared to the controls (Figure 4). With the results of the preliminary studies, concentrations between 10 and 200 μM were selected for the further experiments.

### 2.4. Effect of Micrometam C on LPS-Induced Oxidative Stress in Zebrafish Embryo

**Figure 5:**
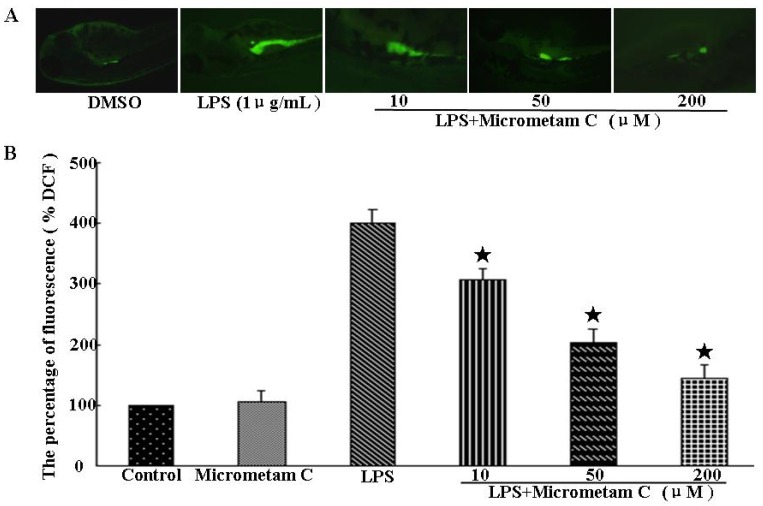
*Micrometam C* inhibits ROS production in zebrafish. (**A**) Representative fluorescence microscopy images. (**B**) The bar chart shows quantitative data. The embryos were treated with LPS in the presence or absence of *micrometam C*. After incubation, the embryos were stained with DCFH-DA and intracellular ROS were detected by spectrofluorometry. *Micrometam C* (10, 50, 200 μM) dose-dependently reduced LPS-induced ROS production (* *p* < 0.01 *vs.* LPS; ANOVA).

Excessive production of ROS is a major contributor to the pathogenesis of inflammation. The protective action of *micrometam C* against LPS-induced oxidative stress was examined in zebrafish embryos *in vivo*. NADPH oxidase-derived ROS production in zebrafish embryos was measured using Dichlorofluorescein diacetate (DCF-DA), a specific dye for intracellular ROS. As shown in Figure 5, the fluorescence intensity was not different between control and micrometam C-treated embryos, indicating that *micrometam C* alone did not affect basal ROS levels. LPS induced a rapid increase in ROS in embryos at 3–4 dpf (Figure 5). In LPS-treated embryos, the relative fluorescence intensity was around 401% ± 21% of the control. In contrast, the relative ROS levels in LPS-stimulated embryos were 306% ± 20%, 202% ± 23% and 144% ± 21% in the presence of *micrometam C* at the concentrations of 10, 50 and 200 μM, respectively. Thus, the present study demonstrates that treatment with *micrometam C* at 10, 50 and 200 μM can reduce LPS-induced ROS.

### 2.5. Micrometam C Restored LPS-Induced Depletion of CAT, GSH and SOD

**Figure 6:**
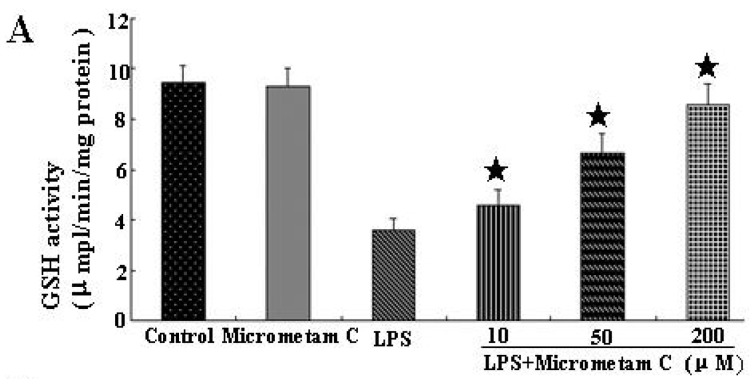

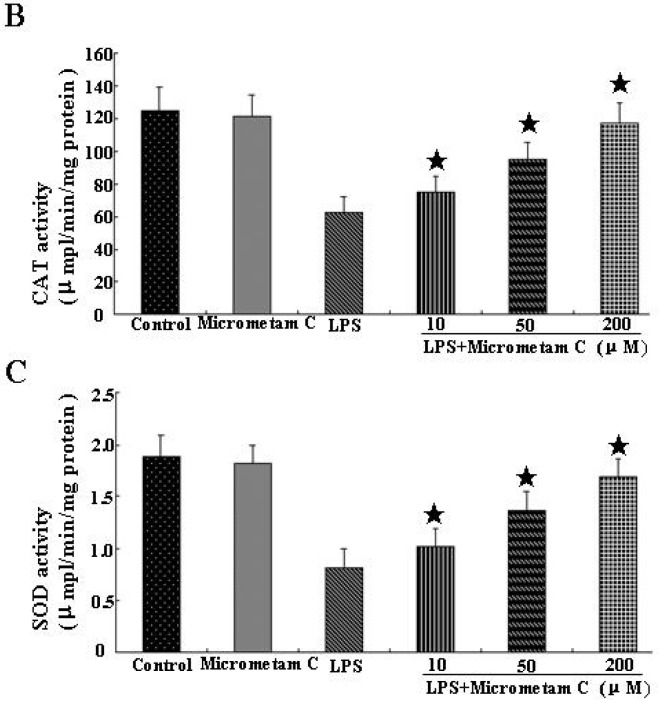
*Micrometam C* restored LPS-induced depletion of GSH, CAT and SOD. (**A**–**C**) Antioxidant enzyme activities in zebrafish after exposure to LPS with or without *micrometam C* supplement. (**A**) GSH; (**B**) Catalase; (**C**) SOD. Data points represent mean activities from more than 25 fishes; error bars represent SEM. * *p* < 0.05 compared to LPS, ANOVA one-way test.

To investigate whether *micrometam C* can restore LPS-induced depletion of endogenous antioxidants, we examined the activities of GSH, CAT and SOD in zebrafish with LPS alone or in the presence of *micrometam C*. As shown in Figure 6, compared with control, *micrometam C* alone did not have any effect on GSH, CAT and SOD. LPS induced a significant reduction in GSH, CAT and SOD. Compared with vehicle treatment, *micrometam C* at 10, 50 and 200 μM significantly restored LPS-induced depletion of CAT and GSH levels in a dose-dependent manner.

### 2.6. Effect of Micrometam C on LPS-Stimulated NADPH Oxidase Activity in Macrophages

**Figure 7:**
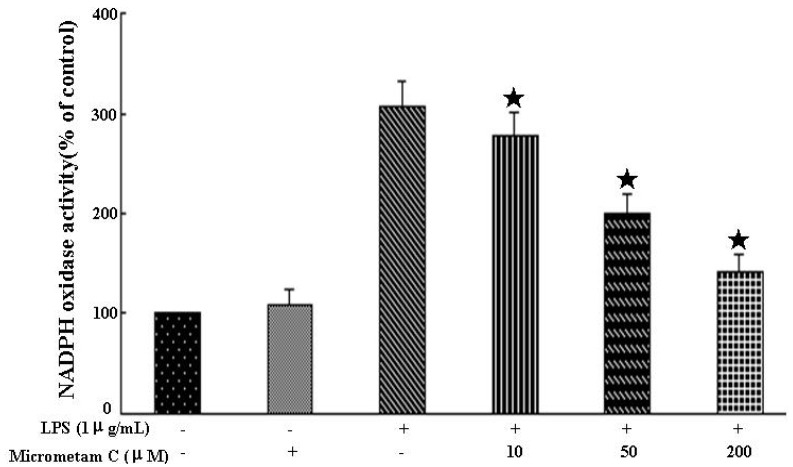
Effect of *micrometam C* pretreatment on NADPH oxidase activity. Cells were preincubated with PBS or *micrometam C* for 40 min, followed by 6 h incubation with or without LPS. The activity of NADPH oxidase was measured using an oxidase activity assay kit. Each value represents the means mean ± SEM (* *p* < 0.01 *vs.* LPS; ANOVA).

As shown in Figure 7, treatment with *micrometam C* alone did not have any effect on NADPH oxidase activity. Treatment with LPS significantly increased NADPH oxidase activity whereas *micrometam C* significantly reduced LPS-induced NADPH oxidase activity. The NADPH oxidase activities were 307.75% ± 22.61% in LPS alone and 277.25% ± 23.84%, 201.13% ± 18.31% and 141.51% ± 18.06% in LPS plus *micrometam C* at the concentrations of 10, 50 and 200 μM, respectively. Thus, the present study demonstrates that treatment with *micrometam C* at 10, 50 and 200 μM can reduce the LPS-induced NADPH oxidase activity.

### 2.7. Anti-Inflammatory Effects of Micrometam C and Its Action on the NF-κB Signaling in Mouse Macrophage Cells in Vitro

**Figure 8:**
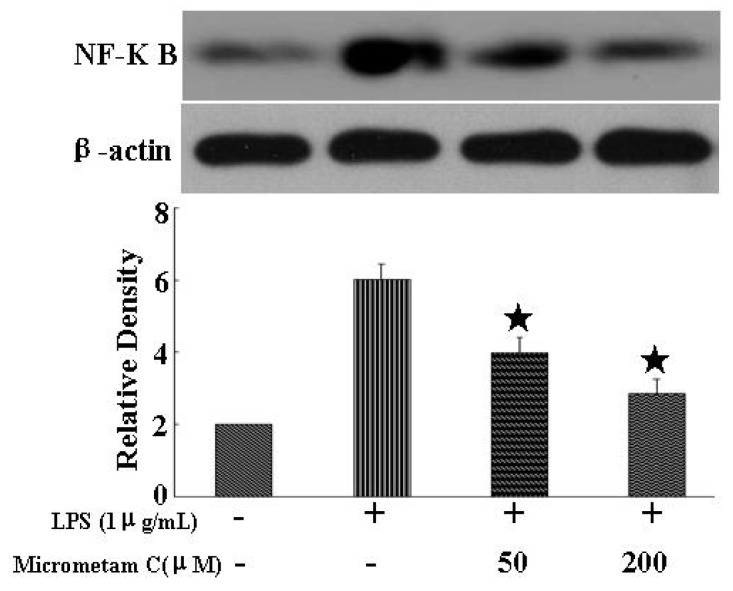
*Micrometam C* attenuated LPS-induced expression of NF-κB, Representative Western blot and densitometric analysis of NF-κB. Treatment with *micrometam C* significantly reduced the expression of NF-κB in a dose-dependent manner. (* *p* < 0.01 *vs.* LPS; ANOVA).

To test whether the zebrafish study can be translated to preclinical studies, we further tested the anti-inflammatory effects of *micrometam C* against LPS-induced inflammation in cultured macrophage cells. NF-κB is considered as a master switch in the regulation of inflammation and immunity. As a transcription factor, NF-κB controls an array of pro-inflammatory genes involved in the inflammatory signaling cascade. Therefore, we examined whether *micrometam C* protect against LPS-induced inflammation through inhibition on NF-κB activation. As shown in Figure 8, *micrometam C* alone did not alter NF-κB phosphorylation whereas treatment with 1 μg/mL LPS for 20 min significant increase the phosphorylation of NF-κB p65. In contrast, pretreatment with 50 and 200 μM *micrometam C* reduced phosphor-p65 levels in LPS-stimulated macrophages. These results suggest that *micrometam C* significantly blocks the NF-κB signaling pathway in LPS-stimulated macrophages through suppression of the phosphorylation of NF-κB p65.

## 3. Experimental Section

### 3.1. Preparation of Micrometam B and Micrometam C

*Micrometam* *B* and *micrometam C* were prepared as previously described [14].

### 3.2. The Isolation of Micrometam B and Micrometam C

*Micrometam B* and *Micrometam C* were isolated and purified by CAS Key Laboratory of Tropical Marine Bio-resources and Ecology, South China Sea Institute of Oceanology, Chinese Academy of Sciences, Guangzhou, China. The purity of the compounds were >98%. Micrometams B–C were dissolved in dimethyl sufoxide (DMSO) and stored at −20 °C until use. The solution form of micrometams B–C was then diluted by PBS to the concentration needed. All reagents were purchased from Sigma (Sigma, Shanghai, China) unless otherwise stated.

### 3.3. Preparation of Inflammation-Induced Zebrafish Model by Tail-Cutting and Application of Micrometam C

Fish Lines—wild-type AB and the transgenic zebrafish line Tg (corola: eGFP), in which both macrophages and neutrophils express eGFP, was kindly provided by the Key Laboratory of Zebrafish Modeling and Drug Screening for Human Diseases of Guangdong Higher Education Institutes, Department of Cell Biology, Southern Medical University, Guangzhou, China and maintained as previously described [15,16]. Zebrafish embryos were generated by natural pair-wise mating of fish that were between 3 and 12 months old. The inflammation assay was conducted essentially as described [17,18]. In brief, 3 dpf Tg (corola: eGFP) larvae were anesthetized in tricaine methanesulfonate solution (Sigma, Shanghai, China) before tail-cutting. After anesthesia, to trigger tail-cutting-induced inflammation, tail of zebrafish larvae was half-cutted and around 50% of tail area was removed. To make wounds as consistently sized as possible, tail-cutting was performed using blade under a dissecting microscope. As soon as tail-cutting was completed, the zebrafish larvae were transferred in the fresh embryo medium and treated with different concentrations of *micrometam C*. Images of zebrafish were captured at 100× magnification using a fluorescent inverted microscope and a CCD camera during the progression of inflammation.

### 3.4. Measurement of the Toxicity of the Isolated Compound

Sample toxicity was determined by means of survival rate and the heart beat rate of the zebrafish embryos. Briefly, the embryos (*n* = 15) were transferred to individual wells of 12-well plates containing 950 μL embryo media from approximately 3 to 4 h post-fertilization (hpf), *micrometam C* was introduced to the embryos up to 24 hpf. The survival rate was measured every day. Then, the zebrafish embryos were rinsed in fresh embryo media. The heart beating rate of both atrium and ventricle was measured at 24 hpf to determine the sample toxicity. Counting and recording of atrial and ventricular contraction were performed for 3 min under the microscope, and the results were presented as the average heart beatingrate per min [19]. The survival rate of zebrafish embryos exposed to *micrometam C* was determined until 5 dpf.

### 3.5. Respiratory Burst Assay in Zebrafish

The respiratory burst assay was performed using zebrafish embryos by measuring oxidation of H2DCFDA to fluorescent DCF [20,21]. Embryos in 96-well microtiter plates (Corning Incorporated, Corning, NY, USA) were pretreated with 10, 50 or 200 μM *micrometam C* for 30 min before LPS incubation. Afterwards, embryos were treated with 1 μg/mL H2DCFDA. The intensity of fluorescence was measured by a Multi-Mode Micro-plate Reader (Molecular Device, Sunnyvale, CA, USA), using excitation and emission filters of 480 ± 10 and 530 ± 10 nm. The intensity of wells containing embryos was normalized to those wells containing culture media only and the results were expressed as the percentage of fluorescence (% DCF) relative to untreated controls.

### 3.6. Endogenous Antioxidant Assay

In order to determine the antioxidant defenses and oxidative stress, we measured the catalase (CAT), glutathione (GSH) and superoxide dismutase (SOD). Initially, the experiments were performed on zebrafish receiving LPS alone or in the presence of *micrometam C* for 24 h (*n* = 6, containing a pool of 20 larvae each). After treatments, the larvae were homogenized in 500 μL of phosphate buffered saline-PBS (pH 7.2–7.4). All samples were centrifuged at 13,500× *g* for 5 min at 4 °C in 1.5 mL tubes and the supernatants were collected for analysis. CAT activity was assessed through the hydrogen peroxide concentration decrease, according to the method described previously [22]. GSH levels were determined by using 5,5′-dithiobis(2-nitrobenzoic acid) (DTNB), according to the method described previously [23]. SOD activity was assayed by measuring the adrenaline auto-oxidation inhibition, according to the method described previously [24]. The results were obtained from three independent experiments performed in triplicate.

### 3.7. Cell Culture

RAW264.7 mouse macrophage cells were obtained from American Type Cultured Collection (Rockville, MD, USA) and cultured in RPMI1640 medium supplemented with 10% heat-inactivated fetal bovine serum, glutamine, and antibiotics at 37 °C under 5% CO_2_.

### 3.8. NADPH Oxidase Activity Assay

NADPH oxidase activity was measured using an assay kit (GENMED, Beijing, China). After a 40 min treatment with *micrometam C* or PBS, cells were stimulated with LPS for 6 h and then harvested. The supernatants were transferred to a tube, to which specific substrate conjugates for oxidase were added. NADPH oxidase activity was determined by spectrophotometry (Thermo Scientific, Rockford, IL, USA) at 340 nm.

### 3.9. Western Immunoblot Analyses

After treatment with compounds and LPS, RAW264.7 macrophages were washed with phosphate buffered saline and harvested with cell lysis. After being centrifuged at 12,000 rpm at 4 °C for 10 min, the protein concentration was determined. After the sample loading buffer was added, protein samples were electrophoresed and then transferred to nitrocellulose membranes (Bio-Rad Laboratories, Hercules, CA, USA). Each membrane was blocked for 1.5 h at room temperature and incubated with specific primary antibodies against mouse anti-NF-kB p65 (1:1000) (Cell Signaling Technology, Danvers, MA, USA).

### 3.10. Statistical Analysis

All data are presented as the mean ± SEM. Differences among test groups were analyzed by ANOVA, using Newman-Keuls multiple comparison test (Prism 4.0, GraphPad Software, Inc., San Diego, CA, USA). A *p* value < 0.05 was considered statistically significant.

## 4. Conclusions

In summary, treatment with *micrometam C* significantly decreased the elevation of ROS levels, increased the CAT, GSH and SOD levels and reduced the inflammation-associated migration of immune cells. Furthermore, *micrometam C* reduced LPS-mediated activation of NADPH oxidase and NF-κB signaling pathway. The present study demonstrates that *micrometam C* protects against LPS-induced inflammation, at least, partially through its antioxidant property.

*Micrometam C* and *micrometam B* were phenethyl cinnamic amides with unsubstituted hydroxyl group and easily activating phenolic hydroxyl groups. *Micrometam C* with a methoxy group at the 7-position of *micrometam B* had a higher antioxidant activity than unmodified *micrometam B*. Thus, the methoxy group at the 7-position of *micrometam* *C* may be benefited to activate 8-hydroxyl group of compound, which is important for its antioxidation activity.
